# Genome sequence of bubaline herpesvirus-1 associated with pustular vulvovaginitis in Indian water buffalo

**DOI:** 10.1128/mra.00889-23

**Published:** 2024-02-20

**Authors:** Sarita Yadav, Ashok Boora, Prasad Thomas, Abinaya Kaliappan, Nisha Verma, Parvina Devi, Nishu Dhaka, Sandip Khurana, Anil Kumar, Sunesh Balhara, Ashok Balhara, Puran Chand Lailer

**Affiliations:** 1 ICAR-Central Institute for Research on Buffaloes, Hisar, Haryana, India; 2 ICAR-Indian Veterinary Research Institute, Bareilly, Uttar Pradesh, India; 3 Indian Council of Agricultural Research, New Delhi, India; DOE Joint Genome Institute, Berkeley, California, USA

**Keywords:** Murrah buffalo, bubaline herpesvirus 1, whole-genome sequencing, pustular vulvovaginitis

## Abstract

We report here the genome sequence of a bubaline herpesvirus 1 isolated from Indian water buffalo. The bubaline herpesvirus 1 strain S102_1 was isolated in 2021 from a Murrah buffalo heifer with clinical presentation of pustular vulvovaginitis.

## ANNOUNCEMENT

Bubaline herpesvirus 1 (BuHV1) is a member of *Varicellovirus* genus of *Alphaherpesvirinae*. Ruminant *Alphaherpesvirinae* subfamily forms a cluster of at least seven recognised viruses, antigenically and genetically related with prototype bovine herpesvirus 1 (BoHV1): BuHV1, bovine herpesvirus 5 (BoHV5), caprine herpesvirus 1, cervid herpesviruses 2, rangiferine herpesvirus 1, and elk herpesvirus 1 (1, 2). The pathogenic mechanisms of BuHV1 in water buffalo is poorly understood, mostly reported with inapparent infections (3–5) and occasionally with reproductive (6, 7) and respiratory clinical manifestations (4, 8). BuHV1 was first isolated in 1972, Australia, from prepuce or penis of buffalo bulls with no lesions (3). Phylogenetic analysis based on partial conserved gene sequences to date has demonstrated that BuHV1 is more closely related to alphaherpesvirus BoHV5 followed by BoHV1 (1, 9).

In June 2021, a suspected herpesvirus infection with clinical manifestation of diffused pustular vulvovaginitis was reported in a 2-year-old Murrah buffalo heifer in the Chhani Badi village, Hanumangarh, Rajasthan, India. The inspected heifer recovered after a few days. In order to identify the causative agent, vaginal lesion sample using vaginal swab was subjected to initial screening by PCR with Glycoprotein gB gene (UL27) primers gB1/gB2 (10) and CR30/CR31 (11). Following PCR confirmation for herpesvirus infection, the virus was isolated from the vaginal swab inducing a cytopathic effect in 25-cm^2^ flask containing monolayer of Madin Darby Bovine Kidney (MDBK) cells at 37°C in the second passage at 96 h post inoculation. For genome sequencing of the BuHV1 S102_1 strain, total genomic DNA was extracted from infected MDBK cells lysate using the DNeasy blood and tissue kit (Qiagen). The paired-end sequencing library was prepared using QIAseq FX DNA Library Kit for Illumina (Catalog: 180479, QIAGEN) from 100 ng of DNA according to the manufacturer’s instructions. The amplified products were then purified using 1 X AMPure XP beads (Catalog: A63881, Beckman Coulter). The raw reads generated using Illumina NovaSeq 6000 (15I bp, 10489990) were indicated with phred quality score above Q20 for 93.37% reads. The reads were processed with fastp (version v0.23.4 ) (12) for removal of adapter and low-quality bases. The quality-trimmed reads were checked for the presence of bovine genome (*Bubalus bubalis*). All the reads which did not align with the bovine genome were mapped to BuHV1 strain b6 reference genome (accession no. NC_043054.1) using a read mapper bwa-mem2 (13) and position-sorted using samtools (14). The consensus sequences were generated using ivar (15) and samtools mpileup (14). All tools were run with default parameters unless otherwise specified. The coverage of consensus sequences was determined using samtools coverage with an overall coverage of 98.21, mean depth 341.41, and mean base quality 35.9. Genome assembly statistics were generated using QUAST ver 5.2 (16). The genome of Indian strain BuHV1 S102_1 represented a scaffold of 137,501 bp involving 17 contigs (>200 b). The genome had a GC content of 74.60%. The BuHV genome was annotated using Prokka v1.14.6 (12). The genome indicated 86 protein-coding genes. The genomic sequence of the BuHV1 S102_1 strain was compared to other complete BuHV1 genomic sequences available in the NCBI database, revealing that the genome sequence was closely related with a nucleotide percentage identity of 97.95% to BuHV1 strain b6 (accession no. NC_043054.1), Australia. To infer the single-nucleotide polymorphism (SNP) variation among both strains, genome alignment was carried out using parsnp ver 1.2 (17). This indicated 1972 SNPs among both strain genomes. The BuHV1 sequence reported here is the first available full BuHV1 genome from the Indian subcontinent and will enrich the existing data of BuHV1 sequences.

## Data Availability

The genome sequence of the Indian BuHV1 strain S102_1 has been deposited in NCBI GenBank under the accession number OQ669137. The raw reads are available under the Sequence Read Archive accession number SRX22329983. The BioProject accession number PRJNA1027138.
